# Changing Culture Around Serious Illness: The VA Life-Sustaining Treatment Decisions Initiative

**DOI:** 10.1093/geroni/igaa057.2708

**Published:** 2020-12-16

**Authors:** Joan Carpenter, Robert Burke

## Abstract

Discussing and documenting goals of care and life-sustaining treatment decisions with seriously ill patients is a widely endorsed practice by healthcare and professional organizations. In 2018, The Veterans Health Administration (VA) initiated a new national policy to standardize such practices, the Life Sustaining Treatment Decisions Initiative (LSTDI), which included a coordinated set of evidence-based strategies and practice standards for conducting, documenting, and supporting high-quality goals of care conversations (GoCCs); staff training to enhance skills in conducting, documenting, and supporting GoCCs; standardized, durable electronic health record tools for documenting patients’ goals and preferences; and monitoring and information technology tools to support implementation and improvement. In this symposium, we will describe the first 20 months of implementing the LSTDI across the VA, the largest integrated healthcare system in the US. The first paper will focus on the factors associated with documentation of a GoCC and treatment preferences. The second paper will present findings describing facilitators and barriers to implementing the LSTDI and identifying factors that promote high rates of LSTDI documentation. The third paper examines patient level outcomes associated with a documented goal of comfort care, specifically the odds of receipt of hospice/palliative care, hospitalization, or ICU admission. This symposium will provide attendees with important information regarding a wide range of individual and system strategies to enhance the care of seriously ill older adults by engaging patients with serious illness in GoCCs and documenting their preferences for treatment in durable, easily accessible notes and orders.

